# Harnessing Microbial Consortia for Efficient Keratinous Biomass Biotransformation

**DOI:** 10.3390/ijms26209898

**Published:** 2025-10-11

**Authors:** Nonso E. Nnolim, Uchechukwu U. Nwodo

**Affiliations:** Patho-Biocatalysis Group (PBG), Department of Biotechnology and Biological Sciences, University of Fort Hare, Private Bag X1314, Alice 5700, South Africa; unwodo@ufh.ac.za

**Keywords:** keratinases, disulfide reductases, microbial consortia, cooperative activity, keratinous biomass, circular economy

## Abstract

Microorganisms exhibit metabolic versatility, which enables their multifaceted application, including in pollutant detoxification, waste recycling, and environmental restoration. Agricultural processing generates substantial byproducts rich in carbon, nitrogen, and sulfur, which require proper handling to mitigate ecological challenges and reduce carbon footprints. The generation of recalcitrant keratinous biomass and its slow degradation in the environment have prompted technological interventions for sustainable solutions. Fundamentally, chemical, thermal and mechanical processing methods have been utilized in managing keratinous waste. These approaches are not only energy-intensive but also yield low-quality products and exacerbate environmental challenges. Multidimensional research on the microbial-assisted conversion of keratinous waste into valuable products, which aligns with circular economy principles, is underway. The biodegradation of keratinous resources has predominantly employed culturable single microbial strains; however, few studies have recently investigated microbial consortia as a promising strategy. The use of microbial consortia leverages the high cultural stability and complementary metabolic pathways of microbes to achieve excellent keratin biodegradation. Therefore, this study examined the latest advancements in transforming keratinous waste into high-quality protein hydrolysates using microbial strains. It detailed various types of microbial consortia and their roles in the valorization of keratinous biomass, while highlighting some knowledge gaps for future studies. The study also explored the role of ancillary microbial enzymes in facilitating the conversion of keratinous biomass into value-added products.

## 1. Introduction

Keratinous biomass is an important bioresource with numerous potential applications when recycled properly. They are largely generated from various agro-processing enterprises, including the poultry industry, barbershops, slaughterhouses, and the leather industry [1]. The accumulation of keratinous waste in the environment presents serious ecological challenges and can lead to odour, air pollution, water contamination, and pathogen transmission due to its slow natural degradation. Effective environmental policy and regulation are crucial for promoting environmental sustainability within a circular economy (CE), which in turn drives sustainable production and consumption [2]. Therefore, researchers are exploring various valorization avenues along the production value chain to reintegrate waste biomass into the economy. Among the methods applied to solubilise keratinous material for potentially recovering valuable keratin from this renewable resource, microbial-aided dismemberment has gained traction due to its cost-effectiveness, ecological friendliness, and high-quality product recovery. Microbial decomposition of keratinous material has been proposed to occur through the inductive secretion of keratinase by microbial cells. Fungal strains, especially dermatophytes, have been naturally endowed with the ability to express keratinolytic enzymes as a means of causing infections in the host [3]. Extracellular keratinase production by bacteria has been proposed as a homeostatic mechanism, particularly in media lacking basic bio-accessible nutrients [4]. Therefore, the production of keratinase by some bacterial strains was adversely affected by the presence of other carbon and nitrogen sources in the cultivation media [5,6]. However, other reports have presented contrary views, suggesting that keratinase production was promoted after the production media were amended with soluble carbon and nitrogen sources [7,8]. It has been proposed that the bioassimilable carbon and nitrogen sources are exhaustively depleted during the acclimatization and initial growth of microbes in keratin-containing production media. The depletion of bio-accessible nutrients further causes the microbes to switch to the insoluble keratinous substrates for nutrients and energy. The presence of keratinous materials in the fermentation media elicits the expression of the inducible keratinase-encoding gene and secretion of keratinolytic enzymes. The extracellular keratinolytic system liberates products, such as amino acids and peptides, from the keratinous polymer through catalytic attacks by endopeptidases, exopeptidases, and oligopeptidases [9,10]. Variations in the amino acid composition of protein hydrolysates from the biodegradation of a particular keratinous material may be caused by the peculiarities and/or metabolic capabilities of the microbial strains. The inefficient conversion of keratinous residues into nutrient-rich hydrolysates by a single strain, due to their usual metabolic capabilities, has necessitated the application of microbial consortia (MC) as an effective strategy. MC have been successfully applied in the saccharification of recalcitrant lignocellulosic biomass [11,12,13], and the degradation of other environmental contaminants [14,15]. Vu and colleagues developed an artificial microbial consortium (AMC) comprising bacterial, fungal and yeast strains, which resulted in an enhanced sugar accumulation during the bioprocessing of wheat bran and wheat straw [12]. Studies on keratinous biomass degradation have shown that MC outperformed the single-strain (SS) strategy based on the analysis profile of the products obtained post-fermentation [16,17]. The enhanced bio-digestion of recalcitrant keratinolytic materials with MC has been attributed to the community-intrinsic characteristics, including environmental fluctuation stability and cooperative actions through metabolic complementation [17,18]. The metagenomic study by Kang and colleagues identified the bacterial community members involved in keratinolysis and provided insights into the genetic basis of keratin decomposition [19]. Keratinous waste recalcitrance and mishandling remain significant challenges to achieving environmental sustainability and resource efficiency until an effective recycling mechanism is developed that promotes a circular economy and new resource creation. Although a few studies have investigated the use of MC for converting keratin materials into bio-accessible, protein-rich hydrolysates, no comprehensive reviews exist on the strategy’s progress, limitations, and prospects. For example, existing literature surveys by researchers in this field have focused on keratinous waste bioconversion, keratinase classification and structure to functional activity [10,20,21,22]. While others have discussed the implications of microbial keratinases in biotechnology and industry [23,24,25,26,27], with the thrust of the aforementioned studies on the application of axenic microbial strains. Therefore, this review assesses the recent advancements in converting keratinous waste into high-quality protein hydrolysates using single microbial strains and MC. It elaborates on different types of MC and their application in keratinous biomass valorization and also identifies some knowledge gaps for future research advancement. Additionally, the study discusses the contribution of ancillary microbial enzymes to ensure the efficient digestion of keratinous biomass.

## 2. Potential Economic Significance of Keratinous Waste

**Figure 1:**
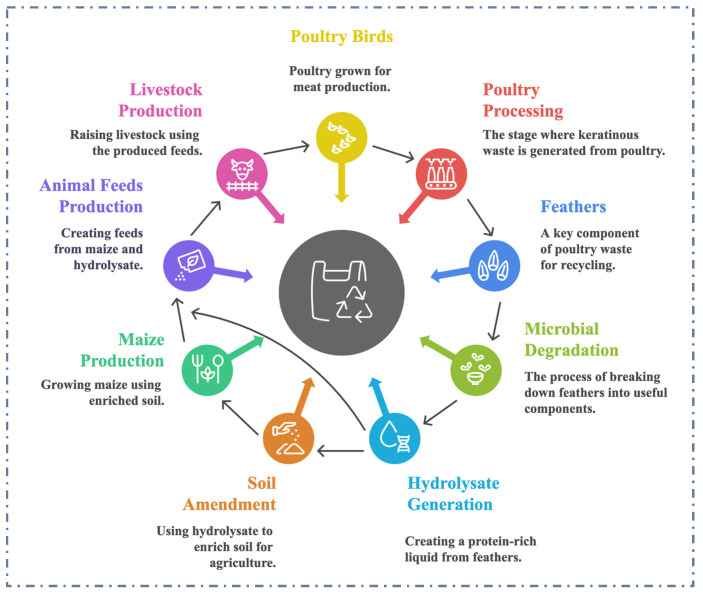
Keratinous biomass generation, recycling and reintegration into the production chain for a sustainable future. The arrows pointing at the core of the spherical structure indicate collective processes promoting a sustainable circular economy.

Keratinous waste, an underutilized and recalcitrant renewable resource, poses a significant environmental challenge, offering both opportunities and challenges for sustainable management and economic exploitation. Keratinous residues are excessively generated as byproducts from commercial enterprises such as slaughterhouses, poultry and leather processing facilities [28]. Inadequate handling or disposal of recalcitrant keratinous waste in the environment poses significant challenges due to its slow decomposition, which leads to air, soil, and water pollution [29]. The development of innovative technologies that can efficiently recycle and reintegrate waste into the economy fosters new business models and generates economic benefits in waste management. A typical pathway showing keratinous waste generation, recycling and reintegration into the production chain for a sustainable and circular economy is presented in Figure 1. Biodegradation of keratinous waste is an innovative process in waste-to-value creation, providing an eco-friendly alternative to traditional disposal methods such as incineration and landfilling [30]. This strategy facilitates the conversion of keratin-rich waste into valuable products, including organic fertilizers, animal feed supplements, biofuel feedstocks, aquaculture feed, bioactive compounds, and biomedical materials [2,31]. Various extraction techniques, including enzymatic, microbial, and chemical processes, can be applied to recover keratin from waste sources such as feathers, hair, and wool [2]. Keratin-based products derived from these processes have diverse applications across industries, including agriculture, cosmetics, pharmaceuticals, bioplastics, textiles, and healthcare [1]. By embracing these sustainable practices, the management of keratinous waste can evolve from a linear to a circular economy model, reducing environmental impact while creating potential revenue streams [2,30,32].

## 3. Traditional Approaches of Keratinous Biomass Valorization

Traditionally, feathers are converted into feather meals through hydrothermal treatment and mechanical processing [33]. Hydrothermal treatment involves subjecting biomass to high-pressure steam, which can modify the physical and chemical structures of the material. This process achieves the weakening of strong intermolecular bonds within keratin, such as disulfide bridges and crystalline structures, thereby enhancing the subsequent solubility and extractability of keratin proteins. The process is carried out at elevated temperatures, typically in the range of 130–220 °C [34]. Hydrothermal treatment combined with mechanical grinding is a method used to degrade keratinous biomass, such as feathers, effectively [35]. This approach leverages the advantages of both high-pressure steam treatment and mechanical size reduction to facilitate the breakdown of rigid keratin structures. However, the products have limited nutrients due to the relative scarcity of soluble keratin proteins, destruction of heat-sensitive nutrients, and modification of others into non-nutritional variants [36].

The hydrolysis of keratinous biomass, such as feathers, using acid and base treatments, is a conventional method for breaking down the rigid keratin structure into useful products [32]. These processes are integral in converting keratin waste into valuable materials, though they come with certain challenges. Acid hydrolysis typically involves using dilute acids, such as sulfuric acid, to break down the keratin. This process is efficient in hydrolyzing the keratin structure, but it can also lead to the degradation of keratin’s valuable proteins and amino acids [32]. High-temperature conditions are often employed, which can accelerate the hydrolysis process but may also increase operational costs and raise environmental concerns due to the production of acidic effluents [37]. In contrast, alkaline hydrolysis, which utilises bases such as sodium hydroxide, targets the breakdown of keratin’s disulfide bonds, primarily responsible for its recalcitrant nature [38]. The alkaline approach often yields better preservation of the amino acid profile compared to acidic methods. It also tends to be less aggressive, thus reducing the extent of protein degradation. However, alkaline hydrolysis can cause issues with chemical waste and requires careful handling of the caustic materials [39]. Table 1 presents a summary of various physical and chemical treatment approaches for valorizing keratinous biomass into valuable products.

Other chemical agents, including sodium bisulfite, sodium dodecyl sulfate, and urea, play a crucial role in the degradation of keratin by breaking down the robust disulfide bonds that characterize keratin’s structure [40]. Sodium sulfide is a commonly used reducing agent in the extraction of keratin from waste chicken feathers. It helps convert feather keratin into eco-friendly bioplastic films, as demonstrated by a study where keratin showed substantial structural retention even after chemical extraction [41].

## 4. Biodecomposition of Keratinous Biomass: Potential Benefits

Keratinous biomass decomposition by microbial candidates has been a field of intense research in recent times due to its potential in a sustainable circular economy. Keratinous waste has been successfully divided into various composite units by bacterial and fungal strains, but with variable degrees of hydrolysis. This variation in the keratinous biomass degradation can be attributed to many factors but largely depends on the microbial peculiarities and metabolic capabilities [42]. The ability of microorganisms to switch between metabolic pathways or express multiple genes in response to their environmental conditions and homeostatic exigencies is crucial for their adaptation and survival [43]. Consequently, keratinolytic microbes adhere to keratin substrates in the cultivation medium and inducibly express keratinolytic enzyme cocktails to digest keratin macromolecular structure for nutrient abstraction, especially where the keratin serves as the primary source of carbon and nitrogen [9,44]. Degradation of keratinous biomass by keratinolytic microorganisms generates hydrolysates that are an excellent source of valuable products, included but not limited to keratinolytic enzymes, amino acids, peptides, and non-protein nitrogen compounds [27,45]. Table 2 shows the potential uses, applications and functional attributes of keratin hydrolysates generated through biotransformation.

Psychrophilic *Penicillium lanosocoeruleum* KSA-55 directed a complete feather hydrolysis through the expression of cold-active keratinolytic enzyme [46]. Similarly, biodegradation of feather waste by *Penicillium oxalicum* AUMC 15084 generated high-quality protein hydrolysates with an abundance of aspartate, glutamic acid, and lysine [47]. Keratinous feather digestion directed by keratinolytic *Chryseobacterium cucumeris* FHN1 yielded feather hydrolysates with 71.46% crude protein, of which 58.97% was amino acids with a high abundance of serine, aspartic acid, glutamic acid and proline [48]. This bacterial strain exhibited a significant keratinase titre of 1030.90 ± 15.42 U/mL after 72 h of incubation. Keratin hydrolysates from microbial (*Streptomyces tanasheiensis* RCM-SSR-6, *Bacillus* sp. RCM-SSR-102) or enzymatic (keratinase HER-102) hydrolysis of feather biomass were a rich source of bioactive peptides [49]. The peptides produced by different bacterial strains and enzymes exhibited variable protein profiles, displayed antioxidant potential, and inhibited the catalytic functions of angiotensin-converting enzyme (ACE), tyrosinase, lipoxygenase, and xanthine oxidase [49]. This observation highlights the economically viable and sustainable production of health-beneficial peptides from relatively cheap feather biomass. Intact feather digestion by *Bacillus licheniformis* WHU yielded bioactive peptides and amino acids [50]. *Trichoderma asperellum*-derived protein hydrolysates from feathers degradation showed bio-stimulating effects by improving the crop growth and productivity [51]. Feed supplementation with feather-derived protein hydrolysates demonstrated significant health benefits in mice, including an extension of intestinal villus height, an elevation of secretory immunoglobulin A levels, increased superoxide dismutase activity in serum, a relatively high abundance of probiotics, and a decline in Proteobacteria and pathogenic species. Similarly, Alahyaribeik and colleagues reported dietary treatment of broiler chicks with keratin-derived mixed bioactive peptides from feather decomposition by *B. licheniformis* [52]. The administration of bioactive peptides via drinking water resulted in significant weight gain, as well as increased villus height and muscle layer thickness, in the treatment group. The researchers also reported a dramatic decrease in serum total cholesterol, triglycerides, and low-density lipoprotein in birds fed mixed bioactive peptides [52]. Additionally, *Bacillus* sp. P45-generated peptides (especially those with molecular weight < 3 kDa) demonstrated an enhanced capacity of radical and peroxyl sequestration [53].

**Table 2:** Potential uses, applications and functional attributes of protein hydrolysates generated through bioconversion of keratinous waste.

Hydrolysate Source	Microbial Strain	Composition	Use	Application Potential	Functional Attributes	References
Chicken feathers	*Bacillus Pumilus* AR57	ND	Bio-stimulant Bio-fertilizer	Agriculture	To promote plant growth, soil fertility and soil microbiota.	[54]
Chicken feathers	*Streptomyces tanashiensis* RCM-SSR-6 *Bacillus* sp. RCM-SSR-102	Bioactive peptides	Antioxidant Enzyme inhibitor	Health	To inhibit angiotensin-converting enzyme (ACE), lipoxygenase, and xanthine oxidase.	[49]
Chicken feathers	*Bacillus licheniformis* AS-S24-1	Nitrogen Amino acids	Organic fertilizer	Agriculture	To enhance the growth of Bengal gram seed germination and promote the soil microbial community.	[55]
Porcine bristles	*Amycolatopsis keratiniphila* D2	Peptides Amino acids	Feed additive	Feed industry	To serve as an alternative protein source in animal feed formulation.	[56]
Chicken feathers	*Bacillus* sp. CL18	ND	Antioxidant Enzyme inhibitor	Health	Displays free radical scavenging activity and inhibits angiotensin I-converting enzyme and dipeptidyl peptidase-IV activities.	[57]
Chicken feathers	*Bacillus subtilis*	Amino acids	Feed additive	Feed industry	To provide an alternative protein source for animal feed.	[58]
Chicken feathers	*Bacillus licheniformis* MW45 *Bacillus paralicheniformis* MW48	Nitrogen	Bio-fertilizer	Agriculture	To promote the germination and growth of spinach.	[59]
Chicken feathers	*Bacillus pumilus* AR57	Amino acids	Microbial media	Biomanufacturing	To serve as an alternative source of peptone for growing microbial strains.	[60]
Chicken feathers	*Bacillus cytotoxicus* LT-1 *Bacillus cytotoxicus* OII-15	ND	Antioxidant	Health	Performs free radical-scavenging activity and displays Fe^3+^ reducing potential.	[61]
Chicken feathers	*Bacillus licheniformis* BBE11-1 *Stenotrophomonas maltophilia* BBE11-1	Amino acids Peptides	Antioxidant Feed additive	Health Feed industry	Demonstrates antioxidant activity and possesses soluble proteins ideal for animal feed formulation.	[62]
Chicken feathers	*Bacillus methylotrophicus* gh1	ND	Bio-fertilizer Bio-stimulant	Agriculture	Increases the fresh and dry weight of lettuce’s shoot and root.	[63]
Chicken feathers	*Streptomyces netropsis* A-ICA *Bacillus subtilis* ALICA	ND	Antioxidant Feed additive	Feed industry	Exhibits an excellent antioxidant property.	[64]
Chicken feathers	*S. maltophilia* K279a *Bacillus cereus* JF70 *Acinetobacter* sp. PD12	Organic carbon Nitrogen Phosphates Potash	Organic fertilizer	Agriculture	Promotes plant growth and development.	[65]
Chicken feathers	*Streptomyces* sp. RCM-SSR-6	Indole-3-acetic acid	Organic fertilizer Phyto-stimulator Biocontrol agent	Agriculture	Enhances the germination of garden pea seed.	[66]
Chicken feathers	*Bacillus* sp. B4	Amino acids	Feed supplement	Feed industry	Serves as a functional supplement for animal production.	[67]
Chicken feathers	*Bacillus safensis* *Aquamicrobium defluvii*	Amino acids Nitrogen	Biofertilizer	Feed industry	promotes the seed germination and vigour index of jute mallow, Cockscomb and Pendant amaranth.	[68]
Goat hair	*Bacillus licheniformis* ER-15	Melanin	Pigment	Cosmetics industry	Sustainable production of personal care products.	[69]
Feathers	*Bacillus amyloliquefaciens* CU33	Amino acids	Feed additive	Feed industry	Promoting the broiler growth by enhancing the duodenal morphology.	[70]
Chicken feathers	*Pedobacter* sp. 3.14.7	Amino acids	Feed supplement	Feed industry	Displayed iron-reducing power, free radical and nitric oxide scavenging activities.	[71]
Feathers Wool	*Trichoderma asperellum*	indole-3-acetic acid Nitrogen	Bio-stimulant Organic fertilizer	Agriculture	Enhancing seed germination and the growth of tomato plants.	[51]
Feathers	*Cladosporium* sp.	Indole-3-acetic acid Ammonium	Bio-fertilizer	Agriculture	Supports the growth performance in tomato seedlings.	[72]

ND: Not determined. Biotransformation of the recalcitrant keratinous biomass results in the generation of bioaccessible products, including amino acids, bioactive peptides, and phytohormones. These products are promising for the sustainable development of the feed, cosmetic, health, and agricultural sectors.

Keratin hydrolysates generated through microbial or enzymatic degradation of keratinous biomass have been utilized as an organic fertilizer to enhance soil fertility [73]. Accordingly, feather hydrolysate from *Stenotrophomonas maltophilia* DHHJ cultivation, with 397.1 mg/L of soluble protein, was found to promote plant growth by 82% or 66% when applied in 2-fold or 4-fold dilutions, respectively [74]. Similarly, protein hydrolysates generated by *Bacillus pumilus* AR57 digestion of chicken feathers promoted the soil fertility and community of the microorganisms present in the soil [54]. Thus, the analysis of the diverse microbiota revealed the following compositions: heterotrophs, 42.23 × 10^6^ CFU/g, nitrogen fixers, 2.45 × 10^4^ CFU/g, phosphate solubilizers, 0.48 × 10^4^ CFU/g, and potassium solubilizers, 0.33 × 10^4^ CFU/g. These soil properties optimally stimulated the growth of maize plants, resulting in increased chlorophyll content (3.7-fold), protein content (1.3-fold), and carbohydrate content (2.3-fold). An enhanced germination and growth of Bengal gram seed and increased soil microbial activity were reported when cultivation soil was amended with hydrolysate from chicken feather degradation by *B. licheniformis* AS-S24-1 [55]. The excellent properties of keratin hydrolysates, including high nitrogen content, bioactive peptides, and amino acids, underscore their promising application prospects in agriculture, health, and livestock production.

## 5. Axenic Culture in Keratin Degradation

**Figure 2:**
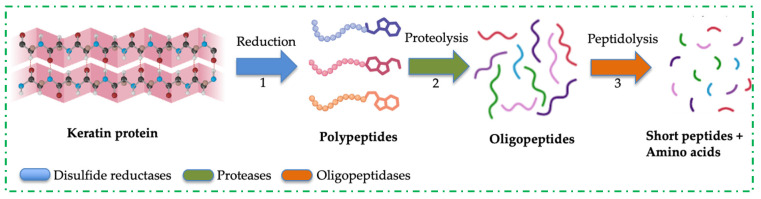
A schematic representation of keratin protein decomposition into soluble products.

An exhaustive list of literature has documented the degradation of keratinous biomass by a single microbial strain [48,75,76,77,78,79]. This functional activity was primarily linked with the extracellular keratinases produced by these microorganisms. Due to the compact-packing nature of keratin protein, microbes have shown variable degradation of this polymer, generating hydrolysates with different constituents of soluble products. The mechanism by which keratin is disassembled into composite units is under intense research; however, sulfitolysis and proteolysis have been proposed as the processes that lead to the complete disintegration of keratin [80]. Sulfitolysis involves the cleavage of disulfide bonds formed due to the oxidation of sulfhydryl groups (-SH) of two cysteine residues. This process is catalysed by disulfide reductases, reductase-like proteins and other microbial cell-bound sulfites [58,81]. Figure 2 shows the bio-decomposition mechanisms of recalcitrant keratin proteins into soluble products. Disulfide bond reduction can also be facilitated by incubating the protein with reducing agents, such as dithiothreitol, 2-mercaptoethanol, glutathione, among other compounds. The inter-chain and intra-chain breakages weaken keratin protein and expose its peptide bonds to protease attacks, resulting in the complete digestion of keratin. Based on the diverse metabolic capabilities of microbial strains, researchers have analysed the keratinous biomass fermentation medium to understand the microbes’ proteolytic enzyme profile during keratin metabolism [9,82]. These studies identified the presence of keratinolytic enzyme cocktails in cultivation medium with keratinous biomass as the main substrate. For example, the study by Yamamura and colleagues was the earliest work that suggested the synergistic action of two *Stenotrophomonas* sp. D-1 enzymes with cooperative actions to digest keratin through disulfide bond reduction and proteolytic attack [83]. It was reported that no keratinolytic activity was observed when the enzymes were singly applied. However, when the enzyme cocktails were engaged, keratinolytic activity increased (>50-fold) compared to the protease application alone [83]. A decade later, a poultry farm-associated *Stenotrophomonas maltophilia* BBE11-1 was recovered and evaluated for keratinolytic potential [84]. Separation techniques and sodium dodecyl sulphate polyacrylamide gel electrophoresis identified three enzymes in the cultivation medium, tagged K1, K2, and K3, with respective molecular weights of 48 kDa, 36 kDa, and 17 kDa. It was discovered that K3 supplementation enhanced the keratinolytic activity of K1 and K2, resulting in significant decomposition of feathers 24 h post-cultivation. It was further established that the synergistic action of K1 and K2 cleared wool cuticle layers without compromising the internal fibres, but the inclusion of K3 in the enzyme cocktails resulted in a noticeable change in the wool structure. The zymography study revealed that K1 and K2 were associated with keratinase activity, while K3 was involved in disulfide bond reduction [84]. In addition, keratinolytic *Pseudomonas stutzeri* K4 was reported to secrete multiple keratinolytic enzymes, with molecular weights ranging from 26 to 76 kDa, during the digestion of chicken feathers in submerged fermentation [85]. The expression of multiple keratinases by strain K4 caused a significant degradation of chicken feather, liberating 784.2 µg/mL of soluble protein. The crude extract, which contains multiple keratinolytic enzymes, also facilitated the dehairing of goat skin, resulting in complete hair removal after 20 h of incubation [85]. *B. licheniformis* ER-15 secreted multiple keratinolytic enzymes during growth on goat hair. The enzyme cocktails successfully dehaired animal hide at 48 h without the addition of lime or sulfite, and also caused significant liberation of melanin from depilated hairs after mild-alkali treatment [69]. Indonesian-originated *Bacillus* sp. MTS was found to disintegrate whole chicken feathers significantly [86]. The analysis of the fermentation medium showed the presence of an enzyme mixture, predominantly keratinase and disulfide reductase. The cooperative action of these enzymes in keratin degradation was tested by using the enzymes singly or in mixture in a reaction flask, and the results indicated that the keratinolytic activity of the enzyme cocktails was greater than that of each enzyme alone, including proteinase K or pure keratinase supplemented with reducing agents [86]. Therefore, the cooperative actions exhibited by the enzyme cocktails profiled so far have revealed the potential mechanisms underlying keratin disassembly. For this reason, the application of MC has become imperative for the effective degradation of keratinous biomass and the generation of protein hydrolysates with improved product profiles.

## 6. Microbial Consortia in Keratinous Biomass Degradation

**Figure 3:**
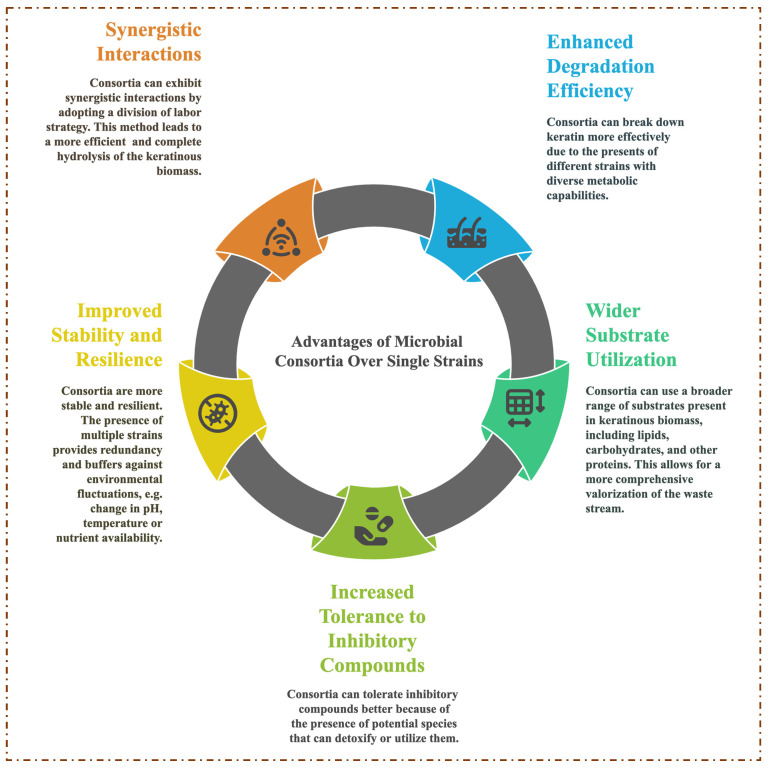
Selected advantages of using microbial consortia in keratinous biomass degradation over a single strain.

Based on the significant successes recorded by axenic microbes expressing multiple keratinolytic enzymes that act synergistically to digest keratin into soluble protein, MC application has been under intense investigation as an alternative approach to recycling keratinous biomass [16]. Figure 3 presents a summary of the advantages of using MC in keratinous biomass degradation over a single microbial strain. MC are generally categorized into two classes based on how the microbial communities are assembled, and they include natural and artificial [87]. Natural microbial consortia (NMC) are the assembly of geo-microbial functional groups that share specific ecological functions and play essential roles in nutrient cycles. AMC are a collection of wild-type microbial strains from the same or different environmental niches that share desired biological functions [88]. The advantages of AMC include a simpler consortium system, compatibility assessment, knowledge of the metabolic capabilities of the strains, and known optimal process conditions of the consortium members [62,89]. The advancement in synthetic biology and bioengineering has further necessitated the development of microorganisms to express specific traits tailored for controlled applications [90]. To this end, AMC have two notable offshoots classified as synthetic and semi-synthetic microbial consortia. Synthetic MC constitute two or more engineered microbial strains with optimized metabolic capabilities designed to execute specific functions. This system has been reported to possess high performance efficiency due to its shared functional activity, which ensures a reduction in metabolic burden [91]. On the other hand, semi-synthetic MC are constructed using a combination of metabolically engineered and wild-type strains. A semi-synthetic consortium demonstrates a high success rate by leveraging the quality contributions of natural diversity and engineered precision [92].

### 6.1. Natural Microbial Consortia in Keratinous Biomass Degradation

MC have demonstrated high efficiency for potential applications in the industrial processing of keratinous waste for various uses. The keratinolytic enzymes expressed by members of MC are expected to cleave keratin polymers at different points, thereby producing protein hydrolysates containing products with diverse molecular weights [93]. These enzymes, due to their varied membership in the MEROPS protease family, including S1, S8, S9, M3, M4, M12, M14, M16, M20, M24, M42, M84, and T3, tend to attack keratin at unique positions, contributing to the high efficiency and performance of MC [10,94,95]. Note that the protease family indicated by S, M, and T represents serine, metallo-, and threonine peptidases, and the number signifies uniqueness within its catalytic type. The formation of a natural microbial consortium with keratinolytic potential involves the successive enrichment of the fermentation medium with keratin substrates, evaluation of keratinolytic activities, and tracking of the consortium composition through molecular studies [16]. Consequently, Kang and co-researchers developed microbial consortium KMCG6 that efficiently hydrolyzed keratinous material with less than 25% undegraded substrate recovered post-fermentation [16]. Time-course analysis of the taxonomic compositions of KMCG6 revealed that the rigorous enrichment process yielded three taxonomically distinct phyla: Bacteroidetes, Proteobacteria, and Firmicutes. The phyla showed no significant changes during the five-day fermentation process, except for fluctuations observed among Alpha-, Beta-, and Gamma-proteobacteria. The compositional analysis at the genus level indicated the presence of eight genera and an unclassified genus. *Chryseobacterium* was the most abundant (>62%), with other genera, *Pseudochrobactrum*, *Acinetobacter*, *Stenotrophomonas*, *Comamonas*, *Buttiauxella*, *Lysinibacillus*, and *Pseudomonas* present in varying degrees [16]. The differential abundance and presence of the isolates throughout the cultivation period underscore the importance of their cooperative existence in facilitating survival and accelerating the decomposition of keratinous substrates. This consortium profile also sheds more light on rare and novel keratinolytic isolates with industrial prospects for further investigation.

**Figure 4:**
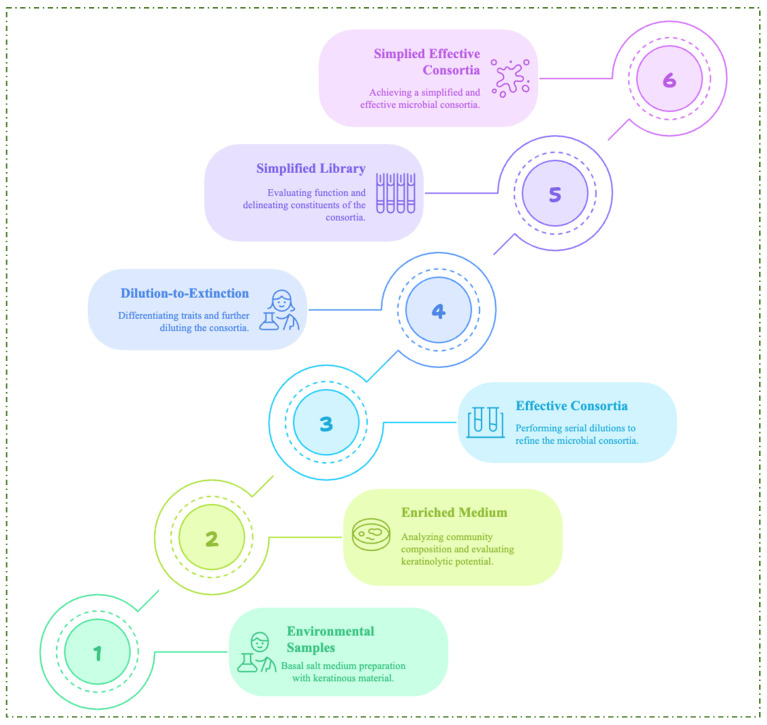
The proposed steps for constructing simplified microbial consortia from environmental samples.

Due to the diversity and complexity of the consortium, an environmental selective pressure undoubtedly occurs during the enrichment process, which enhances the formation of a high-performing microbial consortium for a desired function [18]. However, microbial cheaters with no functional benefits to the consortium may contribute to the complexity of environmental microbial communities. NMC with decreased complexity but enhanced efficiency can lead to an optimized industrial bioprocess. For this reason, the enrichment method and dilution-to-extinction have been employed to create simplified microbial consortia (SMC) of KMCG6, which exhibit excellent keratin degradation capacity and reduced community complexity [18]. The flow chart demonstrating the construction of an efficient SMC is shown in Figure 4. Further study through metagenomic annotation revealed the robust metabolic capabilities of the microbial consortium members [19]. The delineation of metabolic pathways, such as amino acid metabolism, the urea cycle, and disulfide reduction, in the annotated genome of the consortium corroborated the metabolic cooperation required for efficient degradation of keratinous biomass. The researchers highlighted that amino acid utilization for energy requirements and cross-feeding interactions were paramount for maintaining the stability of the consortium [19]. A recent study by Wang and colleagues investigated the dynamics and network of microbial interactions in a consortium enriched with poultry feathers and cultivated at differential temperature conditions [96]. The study revealed that the bacterial species with dominant phyla in all treatment conditions were Firmicutes (56.65%), Actinobacteria (18.13%), Acidobacteria (11.14%) and Proteobacteria (10.35%). The metagenomic studies revealed enzymes such as dipeptidases, aminopeptidases, threonine endopeptidases, metallo-carboxypeptidases, aspartic endopeptidases, and serine endopeptidases coupled with disulfide reductases, confirming the microbial community’s synergism in keratinolysis [96].

### 6.2. Artificial Microbial Consortia in Keratinous Biomass Decomposition

MC have proven more effective than SS in degrading keratinous residues and generating crude protein rich in bio-accessible products. Since NMC are obtained from environmental samples after various evaluation steps, multiple strains in MC may perform similar activity, which culminates in an additive effect. However, the beauty of a microbial consortium lies in the distribution of tasks among its diversified members, where the cooperative activities and complementary traits of the system enhance their efficiency and overall production more than the sum of the individual strains’ contributions. For this reason, researchers have explored alternative approaches to developing effective MC with fewer strains, aiming to achieve excellent degradation of keratinous biomass. One of these methods is the construction of AMC, where single microbial strains meticulously and exhaustively evaluated for a particular potential are carefully combined, allowing for specialization in a cooperative manner to achieve a better output [88]. For example, Saleem and coworkers recently constructed a keratinolytic bacterial consortium, comprising *Bacillus licheniformis* MW45 and *Bacillus paralicheniformis* MW48, for the degradation of poultry feathers [59]. The feather hydrolysate generated was utilized as a biofertilizer for soil fertility amendment. Therefore, it was reported that spinach growth was enhanced more than the control experiment and nitrogen, phosphorus, and potassium (NPK) fertilizer based on the evaluated indices, including the germination percentage, vigour index, leaf numbers, height and weight [59]. Co-cultivation of *Lysinibacillus* MFNC2, *Nocardiopsis* MFNCA and *Micrococcus* MFNCY resulted in 96% hydrolysis of white chicken feathers. In comparison, a maximum of 31% feather degradation was achieved by a single strain under similar optimized process conditions [97]. This bacterial consortium maximally liberated about 635 μg/mL of amino acids at day 5 of feather hydrolysis. Contrarily, the researchers observed that the co-cultivation of *Lysinibacillus* MFNC5, *Nocardiopsis* MFNCA, and Micrococcus MFNCY resulted in the lowest feather degradation (4.08%), which was attributed to the antagonism among the organisms [97]. In another study, Peng and colleagues reported that *B. licheniformis* BBE11-1 and *S. maltophilia* BBE11-1 degraded chicken feathers with percentage hydrolysis of 35.4% and 22.8%, respectively, after 96 h of cultivation [62]. However, after the two bacterial strains were co-cultivated, the degradation rate increased to 55.2% under similar process conditions, suggesting a cooperative action which enhanced the collective capacity of the bacterial strains. When the bacterial strains were used to initiate fermentation in a 3 L fermenter with dissolved oxygen and temperature control, feather digestion increased to 81.8%, resulting in hydrolysates rich in soluble peptides and amino acids [62]. Antagonism among microbial strains has been identified as a crucial factor influencing the effective performance of MC [97]. Consequently, in a recent study by Xia and colleagues, AMC comprising two bacteria (*Priestia aryabhattai* TYB8, *Priestia aryabhattai* YTB20) and one fungal strain (*Bisifusarium keratinophilum* JNF30) was developed, initially evaluated for keratinolytic and potential antagonistic properties [88]. At a 10 g/L optimal medium feather concentration, pH 9.0, and a 2.6% inoculum size, the AMC achieved 74.02% hydrolysis, representing an 11.45% increase compared to the rate recorded in an unoptimized condition. Co-culturing of Armenian geothermal springs-associated *Bacillus borbori* M12 and *Bacillus borbori* M14 improved feather keratin weight loss (>80%) more than a single strain, indicating the superiority of microbial consortium in managing this recalcitrant biomass [98]. Additionally, the co-cultivation of three Streptomyces species on chicken feathers yielded a high quantity of antibiotic agents, including undecylprodigiosin and manumycin A [99]. The detection of some peaks in the co-culture medium, which were absent in the single-strain medium, further underscores the benefits of MC in leveraging the robust metabolic wealth through synergistic interactions.

## 7. Participation of Ancillary Enzymes in Keratinous Biomass Decomposition

Efficient biodegradation of keratinous biomass has been significantly hindered by other compositional components of the macromolecules [40,100]. For example, the study conducted by Tesfaye and colleagues on proximate analysis show that poultry feathers are composed of the following constituents, including crude lipid (0.83%), crude protein (82.36%), crude fibre (2.15%), nitrogen free extract (1.02%), ash (1.49%), and moisture (12.33%) [35]. Feather constituents, such as crude lipid, crude fibre, and nitrogen-free extracts, may have contributed to the challenges encountered by free keratinases or other microbial strains lacking diverse metabolic capabilities during fermentation processes [40]. For this reason, many studies have been conducted using chicken feathers defatted with chemical agents to facilitate the accessibility of the vital bonds in keratinous biomass by hydrolytic enzymes or other treatment methods [101,102,103]. Delipidation of the outer lipid layer of poultry feathers in *Bacillus* sp. 8A6 fermentation medium was observed to expose the keratin’s internal molecular structure for the reduction of disulfide bonds by reductases and metabolic pathway-associated sulfite [95]. A recent study by Shiri and colleagues used lipolytic *Bacillus* sp. TTs1 to defat chicken feathers [104]. The researchers further extracted crude fat from both bio-treated and untreated chicken feathers, and the results indicated respective fat content of 0.92 ± 0.13% and 2.1 ± 0.42%, indicating an eco-friendly and sustainable technology. These observations suggest that in addition to keratinolytic enzymes, microbial strains secrete other enzymes that participate in the structural decomposition of keratinous biomass. Consequently, analysis of the fermentation medium with chicken and goose feathers revealed that *Bacillus paralicheniformis* T7 produced a cocktail of enzymes, including keratinase, protease, collagenase, amylase, xylanase, lipase, and phosphatase [105]. Evaluation of bacteria from geothermal areas in Patagonia, Argentina, for hydrolytic enzymes revealed the production of diverse hydrolytic enzymes in a medium formulated with defatted chicken feathers as the sole carbon and nitrogen source [106]. Among the 30 isolates assessed by the researchers, 16 isolates, distributed among *Geobacillus kaustophilus*, *Bacillus cytotoxicus*, *Bacillus licheniformis*, and *Paenibacillus dentritiformis*, demonstrated extracellular keratinase activity, with differential secretion of other hydrolytic enzymes. Only one isolate showed keratinase activity, along with the production of other hydrolytic enzymes, including protease, amylase, cellulase, inulinase, pectinase, and xylanase, but no esterase production potential [106]. While the remaining keratinolytic bacteria reported by the same authors expressed five or fewer hydrolytic enzymes, suggesting their metabolic diversity. The detection of these enzyme cocktails during bio-digestion of keratinous waste suggests their active or passive participation in compromising different structural components of the biomass for efficient degradation. The structurally compromised biomass is proposed to be further degraded by reductases and completely disassembled by the concerted actions of endo-, exo-, and oligopeptidases from various families, yielding bio-accessible products. This multi-enzyme involvement in keratinous residue decomposition warrants further study.

## 8. Limitations and Prospects

Bioconversion of keratinous waste into valuable products was initiated after the isolation and characterization of *B. licheniformis* PWD-1 over three decades ago. However, research on the application of MC in keratinous biomass valorization is still in its infancy, as most studies have focused on characterizing cultivable single-strain systems. This results in a significant lack of knowledge regarding the dynamics of microbial community interaction during keratinous waste degradation, as well as the participation of microorganisms that are challenging to culture or non-cultivable strains in keratinolysis. A limited report on time course profiling of the consortium during keratinous waste fermentation indicated that there are competitive interactions among the core consortium members, resulting in the proliferation of some bacteria while causing the depletion or extinction of other strains. Therefore, this validates the cruciality of the dilution-to-extinction strategy to the development of SMC with improved efficiency and functionality. A shift in cultural temperature conditions led to enhanced feather hydrolysis, a phenomenon previously reported in NMC; however, the mechanism underlying this observation was not further investigated. The enhanced keratinolytic activity resulting from temperature increase could be attributed to either the microbial community performing optimally at elevated temperatures or the keratinolytic enzymes in the medium being catalytically activated at higher temperatures. The contribution of process conditions in microbial decomposition of keratinous material cannot be overemphasized. Therefore, there is a need to develop a comprehensive optimization approach for NMC, considering that community members may exhibit individual optima based on their unique characteristics. MC have shown great potential in various areas, including environmental and industrial biotechnology. However, microbial community instability is one of the factors that have hindered the efficiency of NMC, especially when dealing with complex substrate transformation. Microbial community instability may arise from various factors, including growth competition, antagonism, metabolite incompatibility, environmental changes, phenotypic drift, and resource heterogeneity. Therefore, new research interests should focus on understanding the growth and metabolic properties of microbial strains, energy requirements, interaction patterns and signalling dynamics within the consortium in order to develop and maintain a robust system with high efficiency. Synthetic biology has led to the development of robust keratinolytic strains with enhanced capabilities. However, there have been no reports on the development of either synthetic or semi-synthetic MC targeted for the valorization of keratinous waste. Therefore, implementing studies using functionally tailored keratinolytic isolates holds immense prospects for industrial keratinous waste recycling and would shed more light on the mechanistic dismemberment of keratin-rich biomass into bioavailable products.

## 9. Conclusions

Keratinous biomass, generated in large quantities by various agro-processing sectors, presents significant environmental concerns. Keratinous biomass, predominantly poultry feathers, has been traditionally converted into valuable products using thermal, physical and chemical approaches. These processing methods yield relatively good products, but the strategies are associated with several ecological and economic challenges. The microbial breakdown of keratinous material through the inducible release of keratinolytic enzymes has emerged as a cost-effective and eco-friendly approach to transforming this waste into valuable products, including organic fertilizers, animal feed additives, bioactive compounds, bioplastic feedstock, and biomedical materials. However, methods using single microbial strains often lead to inconsistent product profiles in the hydrolysates due to inefficient keratin digestion. On the other hand, MC have demonstrated enhanced degradation of recalcitrant keratinous materials through cooperative interactions and metabolic complementation. Both NMC, sourced from environmental samples, and AMC, created with carefully chosen strains, have proven highly effective in the valorization of keratinous waste. Furthermore, ancillary enzymes can play a crucial role in facilitating the modification and complete breakdown of keratinous biomass. Despite progress, research on the use of MC in this field is still in its early stages, with a limited understanding of the dynamics of microbial community interactions and the role of non-cultivable strains in keratinous biomass degradation. Future directions include the development of synthetic or semi-synthetic MC with functionally tailored isolates for industrial keratinous waste recycling towards a sustainable circular economy.

## Figures and Tables

**Table 1:** A summary of some physical and chemical methods of converting keratinous biomass into valuable products.

Treatment Options	Advantages	Disadvantages	References
Acid hydrolysis	It degrades keratinous biomass structure efficiently.	It destroys valuable peptides and amino acids in keratin. It causes the corrosion of equipment. It leads to the generation of hazardous effluents.	[32]
Alkaline hydrolysis	It degrades keratinous waste into valuable hydrolysates.	It results in the generation of toxic chemical waste.	[38]
Reducing agent treatment	It facilitates the extraction of soluble keratin in its native form.	It contributes to the chemical load of the effluents.	[40]
Hydrothermal treatment	It increases the solubility and extractability of keratin proteins from biomass.	It consumes a high amount of energy. It leads to the loss of heat-labile proteins.	[34]
Mechanical grinding	It reduces the particle size of keratinous biomass and can disrupt cell walls and other structural barriers.	It does not improve the bioavailability of the valuable nutrients.	[37]
High-density steam flash-explosion	It destabilizes the inter- and intramolecular bonds, maintaining the structural stability of keratinous biomass. It preserves the structural integrity of soluble keratin.	It is capital-intensive, as the process requires a high investment in energy. It results in the loss of heat-sensitive amino acids.	[34]

The transformation of keratinous waste into valuable products has traditionally been implemented using chemical hydrolysis and physical treatment. These processes are associated with various advantages and disadvantages, as highlighted in the table.

## Data Availability

No new data were created or analyzed in this study. Data sharing is not applicable to this article.

## Abbreviations

The following abbreviations are used in this manuscript:

CE	Circular economy
AMC	Artificial microbial consortia
NMC	Natural microbial consortia
SS	Single strain
MC	Microbial consortia
ACE	Angiotensin-converting enzyme
SMC	Simplified microbial consortia
NPK	Nitrogen phosphorus potassium
